# The Impact of Cooperative Learning Method on the Oral Proficiency of Learners of the Training Program for English Tourist Guides

**DOI:** 10.3389/fpsyg.2022.866863

**Published:** 2022-06-16

**Authors:** Yao Hong, Ling-Ge Chen, Jian-Hao Huang, Yi-Ying Tsai, Te-Yi Chang

**Affiliations:** ^1^Shaoguan University, Shaoguan, China; ^2^Dhurakij Pundit University, Bangkok, Thailand; ^3^National Kaohsiung University of Hospitality and Tourism, Kaohsiung, Taiwan

**Keywords:** cooperative learning method, oral proficiency, quasi-experimental design, English tourist guides, vocational education and training

## Abstract

Oral proficiency is the core element of training courses for English tourist guides. This ability needs to be addressed in training program for English tourist guides. Cooperative learning method is widely used by educators as a teaching method, but rarely used to improve oral proficiency. A quasi-experimental design involving 60 participants was conducted to investigate and examine the effectiveness of cooperative learning method on the oral proficiency of learners in the English tourist guide training program. There were 30 learners in the control group and the experimental group, respectively. The experimental group adopted cooperative learning method, while the control group adopted traditional approach, in both of which pre-test and post-test were conducted. The results of the study showed that the impact of teaching with the cooperative learning method on the oral proficiency of learners of the training program for English tourist guides was higher than that of teaching with the traditional approach. The importance of the cooperative learning method in the training program for English tourist guides is highlighted as a reference for educational institutions.

## Introduction

The tourism industry is booming as the global environment changes and advances. The income generated by tourism has also become an important economic source for many countries (Gidebo, 2021). The most important window of business for the tourism industry is the travel agency, and English tourist guides are an important part of the foreign visitor’s journey (Gani and Damayanti, 2018). One of the advantages that many English learners have in earning a living is the ability to use spoken English. In this case, not only are their employment needs met, but cultural exchange between countries is also facilitated (Youngblood et al., 2021). Saragih et al. (2022) showed that most people in the tourism industry did not speak English to meet the demands of the workplace and that their lack of English proficiency had been shown to be detrimental to global trade. The study by Tran (2021) also pointed out that the training program for English tourist guides was too theoretical but not practical. As a result, how to improve the oral proficiency of learners of the training program for English tourist guides is an important issue at present.

Slavin (1985) pointed out cooperative learning method could be defined as a systematic and structured teaching strategy. In cooperative learning, teachers assign students of different abilities, genders and races to study together in groups, and this teaching strategy is suitable for students of various subjects, disciplines, and ages. With the development of cooperative learning in traditional classrooms, Johnson and Johnson (2018) conducted experiments and collation to develop a systematic cooperative learning method. According to Buchs and Maradan (2021), the learning process in which students work together to achieve group goals in cooperative learning is not only effective in achieving the goal of learning subject knowledge, but also in developing students’ skills of mutual support, cooperation and communication. Hill et al. (2020) also suggested that cooperative learning method increases effective interaction between students, particularly in the listening and speaking aspects of language practice. Compared with individualistic or competitive learning method, cooperative learning method was more effective in promoting social interaction, learner autonomy, and learning success (Shih, 2020; Dzemidzic Kristiansen, 2022). Therefore, the cooperative learning method is a worthwhile pedagogical approach in order to enable learners to learn to become knowledgeable in the English tourist guides domain.

Previous research has shown that teaching style affects learners’ learning outcomes (Watty et al., 2010; Orlov et al., 2021). Darmuki et al. (2018) found that a cooperative learning method had a significant impact on improving the oral language skills of teachers and students within the Indonesian Institute of Language and Literacy Education. Sirisrimangkorn (2021) found that the use of cooperative learning method significantly improved the oral fluency of students. Yavuz and Arslan (2018) found that cooperative learning method had a greater impact on improving vocabulary knowledge, grammar, listening and reading skills of students compared to the traditional approach. Therefore, this study aims to improve the oral proficiency of learners of the training program for English tourist guides through cooperative learning method, which is also one of the directions worth investigating.

English tourist guides are responsible for escorting and servicing inbound tour groups, overseeing the quality of service during the tour and dealing with various emergencies, as well as being partly responsible for business translation during the tour, which requires learners of the training program for English tourist guides to have a high level of oral and verbal skills. Social construction theory believes that learning is the cooperation through consultation between different viewpoints, and the way we obtain knowledge is not based on objective reality, but on the experience sharing of others in the past and present (Burr, 2015). Some studies also point out that social construction theory believes that communication is far from simple description, but constructs the world according to people’s perception and creates knowledge (Retnawati et al., 2018). As the significance of social construction theory has been constantly emphasized, cooperative learning can be used as a teaching method to deepen the understanding of social construction theory (Lenkauskaitė et al., 2020).

Additionally, based on social construction theory, the classroom was the best place for teachers and students to communicate with each other, they interact with each other, meanwhile, when students completed tasks, acquisition of knowledge happened to every learner (Kalina and Powell, 2009). Previous research had revealed the value of social construction theory in enhancing oral proficiency and its value lies in the fact that the classroom provides a social circumstance for learners to communicate and interact with others, and learners’ oral competence can be actively constructed and realized in this social environment (Lantolf and Pavlenko, 1995; Teo, 2016).

This study aimed to explore the impact of cooperative learning method on upgrading the oral proficiency of English tourist guide learners. The results and suggestions of this study can be used as a reference for future education and training institutions to implement the instruction of English tourist guide training program. Therefore, this study is guided by the following research questions: Is there any difference in the impact of the two teaching methods (cooperative learning method and traditional approach) on the oral proficiency of English guide learners?

## Literature

### Theoretical Basis

The basic hypothesis of social construction theory is that learning is a process of negotiation and cooperation with respect to different viewpoints (Burr, 2015). Knowledge has social function and is constructed through understanding and solving problems (Roschelle, 1992). Therefore, the acquisition and organization of knowledge comes from the process of communication, debate, clarification and reconstruction among teachers, students and their peers (Restivo, 2022). Didactic instruction mostly involves the teacher passing on knowledge unilaterally, but social construction theory not only emphasizes on teaching materials to arouse students’ autonomous learning, but also pays special attention to the value of interaction between teachers, students and their peers (Gergen and Wortham, 2001). Both Piaget and Vygotsky contributed greatly to the development of social construction theory. Piaget (1985) emphasized that the construction was based on knowledge building, and that it focused on the construction process of cooperative learning. According to Pigett’s point of view, learners would promote individual cognitive development based on process of discussion. Vygotsky (1986) argued that the fundamental dynamics of individual cognitive development depended on the social and cultural influences and that knowledge was created by individuals and members working together. According to Vygotsky, learners can have better performance if they interact with peers. As a result, cooperative learning method is a teaching method on the basis of the concept of social construction theory.

Learning was an active and ongoing process, education was a way of rebuilding social relationships, and knowledge building occurred when students collaborate, negotiate, and reach consensus in teams (Gordon, 2009; Brinkmann and Tanggaard, 2010). Teachers’ involvement in the design of instructional activities that encourage learners to explain their ideas to each other, discuss differences of viewpoints, and collaborate to solve complex problems, which abide by the concept of social construction theory (Palincsar, 1998). A cooperative group showed positive interdependence, personal responsibility, heterogeneity, shared leadership and so on, when all members of the group participate in and acknowledge their participation in the process of knowledge construction, they recognize the meaning of social existence in the process of participating in knowledge construction, and use this knowledge in their learning process (Krečič and Grmek, 2008; Care et al., 2016). In cooperative learning process, learners had real opportunities to work with each other, share existing experiences, in their learning process, they think critically, operate actively and constructively, they develop motivation and interest in discussing and overcoming uncertainty, while the process of cooperative learning method was defined as a social construction (León-del-Barco et al., 2018). Some researchers have clearly revealed that cooperative learning is an educational design method based on social construction theory (Lenkauskaitė et al., 2020).

Lantolf and Pavlenko (1995) mentioned that the improvement of oral proficiency was not only a cognitive process, but internalized skills through interactive activities, which were realized by active construction in social environment. Thinking and cognition occurred in verbal communication through how the voices shown by others are intertwined with what we say, write and think, emphasizing that the acquisition of speaking skills necessarily comes from interactive learning from the thoughts and language of others and re-expressed interactive behaviors (Teo, 2016). From a social construction theory, using a language to present their thoughts and demonstrate their learning, learners inevitably also improve their oral skills to express, elaborate, explain, synthesize, justify, and revise what they convey (Wilkinson et al., 2017). Language learning was not only passively accepting the “authority” of teachers, or assimilating by “smarter” students who were solidified by traditional knowledge in textbooks, but also through cooperative learning to explore and critique to broaden the learner’s horizons and continuously train oral skills (Johnson, 2006). The improvement of oral proficiency based on cooperative learning method was to encourage learners to integrate the ideas and opinions of participants in the process of oral proficiency training to generate greater negotiation and knowledge construction, to jointly construct knowledge, re-calibrate understanding, and deepen learning (Kohn, 2018). Therefore, this study was based on social construction theory, to explore the effect of cooperative learning method on oral proficiency in English tourist guides training program.

### Cooperative Learning Method

Traditional approach in past studies has mostly referred to the unilateral transfer of knowledge by teachers (Bi et al., 2019; Behmanesh et al., 2020; Jin et al., 2021), but social construction theory not only emphasized that teaching materials should be able to elicit students’ independent learning, but also paid more attention to the value of interaction between teachers and students and peers (Maxim, 2013).

Slavin and Karweit (1985) argued that the cooperative learning method was a structured and systematic teaching strategy. In the cooperative learning method, teachers assigned students to work together in heterogeneous groups based on ability, gender, ethnicity and other criteria. According to Abramczyk and Jurkowski (2020), the cooperative learning method was an activity process which used collaborative teaching strategies to share responsibilities and build positive inter-dependencies among group members. In the collaborative process, group members shared their learning experiences and listened to each other’s ideas to achieve the group’s goals together (Mendo-Lázaro et al., 2018). Therefore, the cooperative learning method is a systematic and structured teaching strategy. The teacher groups learners according to their ability and gender before the lesson is taught. During the learning process, learners are required to collaborate with their group members to complete learning activities, communicate and share ideas in order to achieve the teaching objectives.

There was still some debate about the effectiveness of cooperative learning methods. For example, Johnson and Johnson (2009) pointed out that cooperative learning methods may lead to negative interdependence among students since students hinder each other from achieving common goals. Prosser and Trigwell (2014) argued that cooperative learning method was considered ineffective if students chose to remain passive in cooperative learning, or if students with low social skills had difficulty participating in shared learning. Tadesse and Gillies (2015) believed that students need some time to adapt to cooperative learning; as compared with traditional learning methods, cooperative learning methods take more time to implement, resulting in low teaching efficiency. Loh and Teo (2017) believed that cultural differences affected students’ cooperative learning, and different thought patterns and knowledge systems generated stereotypes and prejudices, leading to unnecessary communication conflicts and affecting the effect of cooperative learning methods. In addition, some studies had pointed out that the cooperative learning method was not suitable for science and engineering disciplines that are regarded as academically rigorous, but more suitable for humanities (Loh and Ang, 2020). However, the cooperative learning method had also been confirmed by many past research results that it can effectively improve students’ English reading comprehension, English writing, and English speaking ability (Ehsan et al., 2019; Awada et al., 2020; Dendup and Onthanee, 2020). Cooperative learning method was not only helpful for students academically, but also very helpful in terms of emotional and social skills, such as appreciation, empathy, and values (Dyson et al., 2021).

Cooperative learning is a teaching method that organize students to learn together and help students interact with each other in the classroom to make learning more effective (Johnson and Johnson, 1993). There were different forms of cooperative learning methods, such as Teams Games Tournaments, Jigsaw, Group Investigation, and Student Team Achievement Divisions (STAD) (Slavin, 1989).

(1) Teams Games Tournaments: quizzes were replaced by weekly competitions, in a competition process, learners were competed with members from other groups to earn points for their group, and the high performing groups were recognized and rewarded for teaching with clearly defined goals (DeVries et al., 1978). The group game competition method was mainly evaluated and encouraged through performance, but the noise caused by the application in the classroom made classroom management difficult, and the subjective judgment of teachers had a greater impact on group performance, making it difficult to ensure fairness (Baydar, 2021).
(2) Jigsaw: arrange students into 4-5 in a group, a leaning task was divided into several parts, and each student was responsible for mastering one of the parts, afterward, students were divided into different groups and had the same learning task, those students learned together and went back to their own group and teach other students (Garcia, 2021). Teaching strategies linked cooperative learning method which students sharing learning tasks with others, this method effectively mobilized the enthusiasm of learners to learn together, but it took a lot of time in the course, and some students were confused because they could understand the classroom tasks (Namaziandost et al., 2020).
(3) Group Investigation: first, teachers make a classroom organization plan, they provided relevant learning topics according to the different situations to each group, and then the group subdivided the topics into different tasks and implemented them on each member; second, the group collected data, discussed together, and prepared to report or presented the learning results to the whole class; finally, teachers or students made an evaluation of each group’s contribution to the class (Asyari et al., 2016). This strategy was particularly prominent in exercising student autonomy, and task relevance was also strong; however, this method required teachers to accompany and follow up students’ progress throughout the process, which required higher teaching ability of teachers, therefore, it was difficult to use this method (Katemba, 2021).
(4) Student Team Achievement Divisions: the aim was to allow students to learn cooperatively with team members of different abilities based on group work, through individual and team performance responsibilities, team members established a positive and mutually dependent cooperative relationship, and student awareness, as students required the efforts of each group member to be successful in the group, those high-achieving students actively helped low-achieving students (Pandiangan, 2019). On the other hand, low-achieving student also studied harder, they got support and encouragement from their classmates, in this way, students of different abilities developed cognition through cooperative learning method, cooperative learning method solved problems in their learning process, and increased their chances of success (Rahmatika, 2019).

To summarize the above viewpoints, Slavin (2013) believed that STAD was suitable for students of different ages, and that STAD was with a wide range of applications and significant implementation effects. Sirisrimangkorn and Suwanthep (2013) found that the implementation of STAD in college students’ English classroom significantly improved students’ oral skills. Ghasemi and Baradaran (2018) confirmed that the use of STAD for English learners in language institutions can significantly improve learners’ speaking skills. Firnanda et al. (2019) disclosed that the use of STAD in high school students’ oral language teaching courses can more effectively improve students’ oral skills. Therefore, this study referred to the previous research, adopts STAD as the form of cooperative learning method in this study, and explored the influence of cooperative learning method on the oral proficiency of English tour guide learners.

Since its development in the 1970s, the cooperative learning method has developed many strategies, the most common of which is STAD. As the content, criteria and assessment used are not very different from traditional teaching methods and are applicable to most subjects (Slavin, 1991), STAD was adopted as a strategy of cooperative learning method in this study. According to Slavin (1991), STAD is designed to allow students to work and learn with their own group members of different abilities through grouping. Through individual and group performance responsibilities, students develop positive and mutually dependent relationships with each other. Students realize that the success of a group requires the efforts of each member of the group, so that students of higher ability can take the initiative to help those of lower ability, thus refining their own learning. On the other hand, students of lower ability can also learn harder with the support and encouragement of their classmates. By working with a more capable group in a similar Zone of Proximal Development (ZPD), they can develop their cognition and increase their chances of success. As a result, students of all abilities can benefit from the cooperative learning method (Rachmawati et al., 2019). Slavin (1991) pointed out that four stages of STAD including (1) whole-class teaching: teacher taught the whole class; (2) group learning: group members discussed and understood the content of the lessons with each other; (3) in-class quizzes: teachers conducted in-class quizzes to assess the oral proficiency of students; (4) group recognition: teachers calculated the progress of the group and recognized the top groups that have made the most progress. As a result, after four stages of instruction, students have a more equal chance of success and progress is more clearly demonstrated.

### Oral Proficiency

Oral proficiency refers to the validity and accuracy of information and knowledge received during language interaction (Haryanti et al., 2021). With regard to oral proficiency, researchers had various view points, such as fluency in spoken English could effectively receive and responded to information, left a good impression on the other party, and interacted effectively and confidently with others (Muslem and Abbas, 2017; Abdullah et al., 2019). Boonkit (2010) pointed out that oral proficiency was one of the skills for effective communication in a second language learning environment, and in English as a Foreign Language (EFL) teaching environment, how to improve oral proficiency is often a key issue in teaching. Blue and Harun (2003) indicated that most foreign tourists could speak English, and English played an important role in tourism industry as a tool for tourism workers to communicate, negotiate and trade with tourists. Bobanovic and Grzinic (2011) pointed out that in the tourism industry, both tourist guides and tourists needed to have good communication to ensure the effectiveness of oral communication, and oral proficiency would be valuable in their work place. Therefore, oral proficiency is an important skill for English tourist guides.

Oral proficiency is an important skill that English learners need to develop and improve (Kehing and Yunus, 2021). Oral proficiency was an important communication method for English tourist guides and tourists, and it was also the teaching goal of English tourist guides training courses (Gani and Damayanti, 2018; Chanwanakul, 2021). Oral proficiency could generally be measured by three indicators, including: (1) Academic achievement referred to the test scores which obtained after speaking training; (2) Number of professional certificates referred to learning in schools or other ability in training places; (3) Off-campus exam referred to the process of participating in various off-campus exams after studying in schools or other professional training places (Liu and Chu, 2010). The participants of this study were English tourist guides learners in educational training institutions. After completing the course training, oral speaking tests were conducted to measure their learning effectiveness. Therefore, this study used the oral speaking test scores as the measure of oral proficiency. In the past, some empirical studies had used oral speaking test scores after learning or training to measure students’ oral proficiency (Karpovich et al., 2021; Khasawneh, 2021; Nuriddinovna, 2021).

### Cooperative Learning Method and Oral Proficiency

Studies found that cooperative learning method has been shown to enhance learning (Retnowati et al., 2017; Carlos Torrego-Seijo et al., 2021). Liu et al. (2018) conducted a study with 36 non-English major undergraduates. The study found that cooperative learning method improved learners’ English listening skills. Rodphotong (2018) conducted a study with 1,471 Year 1 students. The study found that the cooperative learning method had a significant effect on students’ communicative competence in English.

A study of 90 learners in Iran by Namaziandost et al. (2019) found that after using the cooperative learning pedagogy, learners’ oral proficiency improved significantly. A study by Dendup and Onthanee (2020) on 19 fourth-grade students found that the cooperative learning method was more effective in strengthening students’ English communication skills than traditional teaching methods. A study by Köroǧlu (2021) of 52 first-year college students in Turkey showed that the cooperative learning method was very effective in cultivating and developing the spoken language of foreign language learners. Haryanti et al. (2021) indicated the survey of 64 students showed that the cooperative learning method was effective in promoting students’ engagement in language learning activities and oral proficiency. To conclude, cooperative learning method is an optimal approach to enhance effective in helping students to learn basic language skills.

In summary, most researches indicated that the cooperative learning method had a significant impact on learners’ oral proficiency. There is a lack of research on the effectiveness of cooperative learning method in educational institutions, especially in the filed of training English tourist guides. Therefore, this study proposed a hypothesis that the experimental group receiving cooperative learning method could have significantly higher post-test scores in oral proficiency test than the control group.

## Materials and Methods

### Participants and Ethics Approval Standards

This study involved 60 learners from the same English tourist guide training institution. There were 30 learners in each of the two classes. One class was the experimental group and the other was the control group. The experimental group consisted of 23 (76.7%) male students and 7 (23.3%) female students. The control group consisted of 24 (80.0%) male students and 6 (20.0%) female students.

In addition, there are 20 people (66.7%) aged 20-30 years old in experimental group, 7 people (23.3%) aged 31-40 years old, and 3 people (10.0%) aged 41 years and above. There are 20 people (66.7%) aged 20-30, 9 people (30.0%) ranging from 31 to 40, and 1 person (3.3%) above 41 years old. 20 people (66.7%) in the experimental group did not have tourist guide licenses, 10 people (33.3%) had tourist guide working experience, 20 people (66.7%) in the control group also had no working experience, and 10 people (33.3%) had working experience of tourist guide as showed in Table 1.

**TABLE 1:** Basic information of participants.

Basic information	Group	Experimental group (*n* = 30)		Control group (*n* = 30)	
			
		Frequency	Percentage	Frequency	Percentage
Gender	Male	23	76.7	24	80.0
	Female	7	23.3	6	20.0
Age	20–30	20	66.7	20	66.7
	31–40	7	23.3	9	30
	Above 41	3	10	1	3.3
Background	Without tourist guides working experience	20	66.7	20	66.7
	With tourist guides working experience	10	33.3	10	33.3

In accordance with the Declaration of Helsinki (Goodyear et al., 2007), this study was conducted under the condition that all participants voluntarily cooperated and signed an informed consent form, after fully considering the privacy and willing of the participants, participants were allowed to refuse or joint this research.

### Research Design

**FIGURE 1:**
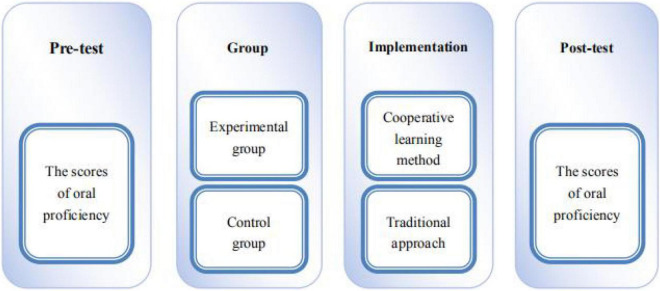
Research design of the study.

This study adopted the quasi-experimental design and selected learners from an English tourist guides training institution as the participants of the study by means of purposive sampling. The participants were divided into an experimental group and a control group. Both groups of learners were given a pre-test of oral proficiency, and then the experimental group was taught using the cooperative learning method, while the control group was taught using the traditional approach. At the end of the course, post-tests were administered to the experimental group and control group, and the research design is shown in Figure 1.

### Independent Variable

The independent variable in this study is the experimental treatment of the cooperative learning method. The experimental treatment is divided into two parts: learners who received cooperative learning method were considered as the experimental group; learners who received traditional approach were considered as the control group.

### Dependent Variable

The dependent variable in this study is oral proficiency, in other words, the oral proficiency scores of the learners of the training program for English tourist guides.

### Research Implementation Procedure

In this study, the experimental group used the STAD of the cooperative learning method. The duration of instruction was 12 weeks, with one 60-min lesson, two lessons a week, for a total of 120 min. Learners in the experimental group and control group are required to complete a pre-test of their oral proficiency before their first lesson and a post-test of their oral proficiency at the end of the twelfth week of the course. Both the experimental group and the control group were taught the same 12 topics from English for International Tourism, while 12 topics are followed the same teaching objectives, teaching materials and in-class tests.

To fit the nature of this oral training class, teachers chose “English for International Tourism” (Dubicka and O’Keeffe, 2013) as a textbook. This version is divided into three series, which are: pre-intermediate, intermediate, upper intermediate. This study is a pre-intermediate, and this version is a language course designed for learners interested in the tourism industry with the goals of developing learners to express their opinions in English, describing familiar people, things, places, things and participating in simple discussions, and even building confidence in English communication (Sarem et al., 2013). Daoud and Celce-Murcia (1979) provided an assessment checklist that had been widely used for textbook assessment and consists of five indicators, including: (a) subject matter, (b) vocabulary and structures, (c) exercises, (d) illustrations, and finally (e) physical make-up. Sarem et al. (2013) critically examined “English for International Tourism” by adopting 5 indicators of the above evaluation list, and results showed that “English for International Tourism” was used as a textbook to teach motivated students about Tourism in terms of good content validity.

**FIGURE 2:**
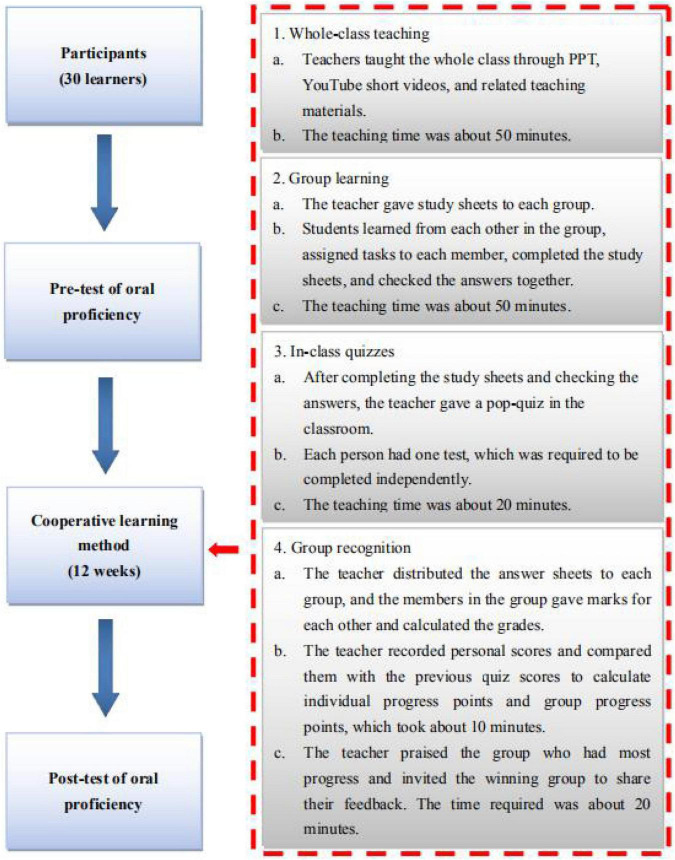
Implementation procedures of the experimental group.

In the control group, the teacher first taught the class the content based on textbook, a combination of handouts, PowerPoint presentations and short videos, which took 70 min. Afterward, learners were then required to complete classroom sheets and check their answers independently, which took 20 min. Then, the teacher then arranged a 20-min quiz in class, which took 20 min. Finally, the teacher announced the answers and the learners corrected each other’s answers and the test results were recorded by the teacher, which took 10 min. In the experimental group, the STAD cooperative learning method was used for all 12 weeks of the course. Before the experiments began, teachers were required to prepare a lesson plan and teaching aids based on STAD. In addition, learners were graded into five levels - A, B, C, D, and E - based on their pre-test scores of high, medium-high, medium, medium-low, and low oral proficiency. The groups are grouped according to the principle of heterogeneous grouping. In the classroom, the group members sat together in a circle. A and E sat side by side, while B, C and D sat interspersed. As shown in Figure 2.

### Assessment Tool

The assessment tools for this study used test questions from the “English for International Tourism” textbook edited by Dubicka and O’Keeffe (2013). The pre-test (please refer to Supplementary Appendix A) and post-test (please refer to Supplementary Appendix B) of the experimental group and the control group were the same in the test content, including: reading and comprehension tests. The learner’s learning task was to improve the oral proficiency. The purpose was to check the level of the learner’s oral proficiency, and learners needed to complete the test by reading a piece of spoken English aloud and answering some comprehension questions on the reading in English.

Validity and reliability were achieved through the collaboration of 2 raters. Both evaluators are university professors with more than 30 years of experience in English-speaking guide training courses, and both have doctorate degrees in tourism management. In order to examine the stability and consistency of the pre-test and post-test scores of the 2 raters, we used Pearson’s correlation coefficient to calculate test-retest reliability. Test-retest reliability for the first rater was 0.976, *p* < 0.001, and test-retest reliability for the second rater was 0.970, *p* < 0.001, indicating good test-retest reliability (Rousson et al., 2002). According to the evaluation of Taiwan English Tourist Guide Exam (Ministry of Examination R.O.C., 2013), 2 evaluators used 3 assessment criteria to assess the learners’ oral proficiency, including: foreign language expression ability, pronunciation and intonation, talent and insight. These 3 evaluation criteria were divided into 6 quality levels, and each quality level was clearly defined, as shown in Table 2.

**TABLE 2:** Definitions of grading criteria.

Level	Foreign language expression ability	Pronunciation and intonation	Talent and insight
0	Silent and no responses to any questions		
1	A very small bank of vocabulary and incorrect grammar	Too slow speaking rate, unclear pronunciation and incorrect intonation with many pauses	Unable to express perspectives completely
2	Limited amount of vocabulary and many grammatical errors	Slow speaking rate, poor pronunciation and disfluency of intonation with few pauses	Unable to express perspectives correctly
3	Inadequate vocabulary, able to use correct grammar frequently, and some grammatical errors	Slow speaking rate, understandable pronunciation while bizarre intonation	Able to express perspectives at least one idea with a few errors
4	Adequate amount of vocabulary, able to use correct grammar usually and few errors	Moderate speech rate, clear pronunciation and correct intonation	Able to express many perspectives with few errors
5	A large bank of vocabulary, and always able to use grammar correctly	Fluent speech, clear and precise pronunciation and very fluent intonation	Ability to express plenty of perspectives correctly and explain arguments in detail

According to the suggestions of the 2 evaluators, foreign language expression ability was 60 points, pronunciation and intonation was 20 points, talent and insight was another 20 points, and the total score was 100 points. Quality levels (from 0 to 5) are converted into fractions. Except for Level 0, each level of foreign language expression ability is worth 12 points, and each level of Pronunciation and intonation, talent and insight was 4 points for each items, total score was 100 points, the higher scores students obtained, the better oral proficiency they attained overall. According to the evaluation of Taiwan English Tourist Guide Exam (Ministry of Examination R.O.C., 2013), the participants who could pass the exam should have to get above 60 points. When pass mark is converted to quality assessment level in this study, the passing grade must be greater than level 3. Table 3 shows the distribution of score allocation.

**TABLE 3:** Grading criteria.

	Level 0	Level 1	Level 2	Level 3	Level 4	Level 5
Foreign language expression ability (60 points)	0	1-12	13-24	25-36	37-48	49-60
Pronunciation and intonation (20 points)	0	1-4	5-8	9-12	13-16	17-20
Talent and insight (20 points)	0	1-4	5-8	9-12	13-16	17-20

After a 12-week English tour guide training course, two evaluators evaluated the oral proficiency of 30 learners in the experimental group. To ensure that the two assessors’ scores were consistent, this study used Spearman’s correlation coefficient to calculate the inter-rater relationship. Reliability, with a correlation coefficient was.928, *p* < 0.001, conforming to the criteria for inter-rater reliability greater than 0.90 (Miles and Huberman, 1994). Therefore, the inter-rater reliability was good.

### Data Analysis Method

In this study, statistics and analyses of various data were carried out using IBM SPSS Statistics Version 22.0. Descriptive statistics were used to describe means and standard deviations of oral proficiency. Next, the analysis of covariance (ANCOVA) was used to compare post-test scores of the verbal ability of the two research groups after 12 weeks of guidance, and the *post hoc* comparison was used to further verify whether the two research groups had achieved significant differences in the post-test.

## Results

This study was a quasi-experimental study, so ANCOVA was used, where the pre-test scores were considered as the covariate, the groups of research participants as independent variables, and the post-test scores as dependent variables for ANCOVA. Prior to ANCOVA, “the test of homogeneity of within-group regression coefficient” was performed. After the homogeneity test showed that the data were suitable, ANCOVA was performed. The results of the analysis were shown below.

As shown in Table 4, the *F*-value for homogeneity of regression coefficient did not reach significant levels (*F* = 0.890, *p* > 0.05), which was consistent with the basic assumption of homogeneity of regression coefficients within groups, and therefore ANCOVA was continued.

**TABLE 4:** Summary of the test of homogeneity of within-group regression coefficient for oral proficiency.

Source	Sum of squares	Degree of freedom	Mean sum of squares	*F*	*P*
Group	0.024	1	0.024	0.013	0.909
Pre-test	814.974	1	814.974	436.514	0.000
Group*Pre-test	0.036	1	0.036	0.019	0.890
Error	104.552	56	1.867		
Corrected total	5,164.433	59			

As shown in Table 5, the main effect of the group reached critical significance in the ANCOVA for oral proficiency (*F* = 6.325, *p* < 0.05). There was a trend for the experimental group to have a larger mean (*M* = 68.410) than the control group (*M* = 66.663) after post-test correction (see Table 6).

**TABLE 5:** Summary of *post hoc* comparison for oral proficiency.

Source	Sum of squares	Degree of freedom	Mean sum of squares	*F*	*p*	LSD *post hoc* test
Pre-test	1,744.578	1	1,744.578	950.78	0.000	Experimental group >Control group
Group	11.605	1	11.605	6.325	0.015	
Error	104.589	57	1.835			
Corrected total	5,164.433	59				

**TABLE 6:** Description of the participants in the pre-test and post-test of the oral proficiency.

Group	Pre-test		Post-test		Post-test (corrected)	
			
	*M*	*SD*	*M*	*SD*	*M*	*SD*
Experimental group (*n* = 30)	72.967	7.550	74.800	7.289	68.410	0.330
Control group (*n* = 30)	58.867	3.003	59.933	3.261	66.663	0.330

## Discussion

The purpose of this study was to examine the development of oral proficiency among English tourist guide learners after receiving cooperative learning method. According to the results of ANCOVA, the oral proficiency of the experimental group was significantly higher than that of the control group. The result showed that cooperative learning method effectively increase learner’s oral proficiency.

First, the results of this study confirmed the applicability of social construction theory in explaining the cooperative learning method in the oral proficiency training of English tour guide learners. Since the basic assumption of social construction theory was that learning was through negotiated cooperation between different viewpoints (Restivo, 2022), cooperative learning method allowed learners to undergo a process of social construction, such as participating in teamwork (Gordon, 2009), communication and interaction (Krečič and Grmek, 2008), negotiation and discussion (Kohn, 2018), from others’ viewpoints and generating one’ s own viewpoints (Teo, 2016), collaboratively discuss and find answers (Wilkinson et al., 2017). All of the above processes cultivated and improved learners’ oral proficiency. Therefore, social construction theory had important value for cooperative learning method in oral proficiency training.

Second, Many researchers had also affirmed the effectiveness of cooperative learning method in promoting oral proficiency. The results of this study were consistent with those of some empirical studies. For example, the interactive behavior of cooperative learning method had a positive impact on students’ learning outcomes and language skills (Cabrera, 2018; Darmuki et al., 2018), and cooperative learning methods allowed students to easily use English to interact with classmates, significantly improving students oral ability (Calderón et al., 2011; Dendup and Onthanee, 2020). The possible reason was that the cooperative learning method stimulated learners to show a higher enthusiasm for learning in the classroom question-and-answer session and classroom group discussion (Alrayah, 2018), and high-achieving students felt more fulfilled in the process of helping low-achieving students (Ghaith, 2001), providing learners with the freedom to share their ideas actively and positively (Suamuang and Suksakulchai, 2020). Therefore, the results of this study provided empirical support for the cooperative learning method to help learners of the training program for English tourist guides.

However, the results of this study differed from some previous studies. Some researchers believed that cooperative learning methods were ineffective and even gave students some negative learning effects. For example, the difficulty of participating in shared learning (Prosser and Trigwell, 2014), the difficulty was that students needed more time to get used to cooperative learning (Tadesse and Gillies, 2015), the conflict of cultural differences (Loh and Teo, 2017), the conflict of disciplinary differences (Loh and Ang, 2020). This may be due to the differences background of participants. The above studies all adopted college students as participants. However, English tourist guides learners are more extroverted and proactive than college students as they have occupational character traits, they were less likely to encounter the above learning difficulties or conflicts. There were still some limitations in the implementation of the cooperative learning method, which was worth further research.

## Conclusion

This study revealed the effect of cooperative learning method on English tourist guide learners’ oral proficiency. The results showed that the English tourist guide learners in the experimental group had better oral proficiency than the control group after the cooperative learning method was implemented. That is, the cooperative learning method resulted in better teaching outcomes, which helped to improve the oral proficiency of the learners in the training program for English tourist guides. This result was in line with the findings of Chan et al. (2019), which showed that the cooperative learning method had a positive impact on oral proficiency. The possible reason was that learning was a cognitive process. This process was aimed to meet the psychological needs of learners. As learning behavior was social-able, the process of learning was accompanied by lots interactions and communications.

### Theoretical Contributions

The results of this study have demonstrated the positive effect of the cooperative learning method on the oral proficiency of English tourist guide learners. This research was based on the social construction theory, this study verified that cooperative learning method could be an effective teaching method and has a positive effect on the academic research of education field. Previous literature has emphasized the importance of cooperative learning method in classroom learning for students of all ages (Namaziandost et al., 2019; Dendup and Onthanee, 2020; Haryanti et al., 2021; Köroǧlu, 2021), compared with previous literature, this study focuses on the training process of English tourist guide learners and integrates cooperative learning method into the curriculum. Empirical tests confirmed that cooperative learning method effectively could improve English tourist guide learner’s oral proficiency. That means cooperative learning method play a role in English tourist guide training course. To sum up, this study proposed a training model of cooperative learning method for English tourist guide learners’ oral proficiency, which provided a new perspective for educators and training institutions. This research also broadened the application of cooperative learning method in different education field.

### Practical Implications

This study found that the STAD had a significant impact on the oral proficiency of learners of the training program for English tourist guides. It is therefore recommended that training units develop teaching plans and programs based on the STAD to provide guidance to teachers in the delivery of lessons. It is recommended that flexible and interesting teaching methods be used during the whole-class teaching phase with the use of multimedia technology. In the group learning stage, teachers should play the role of supervisor and coordinator to create a harmonious and interactive learning atmosphere. At the in-class quizzes stage, teachers should assess learners’ achievements against strict criteria. During the group recognition stage, teachers should start to objectively evaluate the groups that have made more progress according to their results in order to increase the group’s sense of honor. The integration of the cooperative learning method into the teaching of English as a foreign language would enable teachers to better grasp the teaching process and methodology of the cooperative learning method and to better achieve the teaching objectives in order to enhance the oral proficiency of learners.

### Limitations and Future Directions

This study found that the cooperative learning method had a significant impact on the oral proficiency of learners of the English tourist guides training program. However, according to the evaluation of Taiwan English Tourist Guide Exam (Ministry of Examination R.O.C., 2013), those people attending this exam should have to get above 60 points. Therefore, this study only focused on probing into the influence of cooperative learning method on the total scores of oral proficiency test of English tourist guide learners instead of analyzing the 3 assessment criteria (foreign language expression ability, pronunciation and intonation, talent and insight). Future studies can further explore different assessment criteria or assessment tools.

The participants of this study were all learners of the training program for English tourist guides in the training institutions. Conclusions could only be inferred at the level of the training providers. Due to the constraints of the researcher’s resources, the 12-week experiment was conducted on two classes at the English tourist guides training institution. Both the experimental group and control group consisted of 30 students. It is suggested that future studies could extend the duration of the teaching experiment, increase the sample size, combine different cooperative learning method, all of which are worthy of further study and research by future researchers.

In this study, quantitative analysis was used to investigate whether there was a statistically significant difference between the experimental group and the control group in terms of their oral proficiency with the cooperative learning method. The reasons for the differences were mostly based on the observations and inferences of the researchers. The factors affecting the learners were not explored in greater depth. It is suggested that future research could include qualitative interviews to clarify the reasons why learners improve oral proficiency significantly after experiencing the cooperative learning method.

## Data Availability Statement

The raw data supporting the conclusions of this article will be made available by the authors, without undue reservation.

## Ethics Statement

The studies involving human participants were reviewed and approved by Shaoguan University. The patients/participants provided their written informed consent to participate in this study. Written informed consent was obtained from the individual(s) for the publication of any potentially identifiable images or data included in this article.

## Author Contributions

T-YC and Y-YT contributed to the conception of the study. Y-YT collected and organized the data. YH and L-GC contributed significantly to the analysis and manuscript preparation, performed the data analyses, and wrote the manuscript. J-HH helped perform the analysis with constructive discussions. YH was responsible for the overall project. All authors contributed to the article and approved the submitted version.

## Conflict of Interest

The authors declare that the research was conducted in the absence of any commercial or financial relationships that could be construed as a potential conflict of interest.

## Publisher’s Note

All claims expressed in this article are solely those of the authors and do not necessarily represent those of their affiliated organizations, or those of the publisher, the editors and the reviewers. Any product that may be evaluated in this article, or claim that may be made by its manufacturer, is not guaranteed or endorsed by the publisher.
