# Supplementary material for: The Impact of Cooperative Learning Method on the Oral Proficiency of Learners of the Training Program for English Tourist Guides
Source: Front Psychol. 2022 Jun 16;13:866863. doi: 10.3389/fpsyg.2022.866863 (PMC9245035; doi:10.3389/fpsyg.2022.866863)
Supplement: Supplementary file 1 [file Data_Sheet_1.pdf]

# Appendix A

**Paragraph reading:**

When I think of a popular place in my city, I think of "Trailside Park." This is a very large park, and it has many different parts for different activities. Of course, families bring their children to the park to have fun on the playground area. Many people bring their dogs to walk and play in the park. Also, a lot of people jog or walk through the park or go to yoga classes. One large area of the park is only for team sports, so people can play soccer, baseball, and basketball. Some parts of Trailside Park have special uses. For example, there are small restaurants and cafes and places for theater and music performances. There is free wi-fi, so some people bring their laptops and sit on the benches to work and study. In my opinion, places like Trailside Park should be in every city. I think Trailside Park is popular because it is an interesting and beautiful place with all types of people. It is also possible to do many things at the park in one day. For example, I can take a yoga class in the morning and then have lunch at a café. Any day of the week, there is something to do at Trailside Park.

**Comprehension questions:**

1. What is your favorite food? What food do you not like?
2. What two places would you like to visit on your next holiday?
3. What are two things you usually do on weekends?
4. Describe a place in your city or in a city that you know well that is used by many people for many different purposes. Who uses this space and for which purposes?
5. Why do you think this place is popular?

## Appendix B

**Paragraph reading:**

Hilton hotels & Resorts is an international chain of full-service hotels and resorts it is the flagship brand of Hilton Worldwide. As the global in hospitality, Hilton Hotels & Resorts currently serves guests worldwide with more than 550 properties in 80 countries and across six continents Hilton Hotels are recognized around the world, offering guests and customers the fines accommodations, service, amenities, and value for business or leisure.

The original company was founded in 1919 by Conrad Hilton. Conrad Hilton (1887-1979) was born in San Antonio. He was determined to maximize ever square foot of hotel space and serve the hotel guest with a mind of "take me to the Hilton." Mr. Hilton bought his first hotel, the Mobley Hotel, in Cisco, Texas in 1919. The company is now owned by Hilton Worldwide. Hilton hotels are wither owned by, managed by, or franchised to independent operators by Hilton worldwide. In late 2010, Hilton Worldwide announced a name change of the Hilton Hotels brand to Hilton Hotels & Resort along with a new logo design as part of re-branding effort for the flagship brand.

**Comprehension questions:**

1. What basic information do you know about Hilton Hotels &Resorts in terms of its identity, status in hospitality, foundation and its maxim?
2. Who own Hilton Hotels & Resorts now and why was the Hilton Hotels brand changed to Hilton Hotels &Resorts along with a new logo design?
3. What is Hilton's marketing emphasis and what do you think of the accommodations and food and beverage provided by Holton Hotels?
